# Physicochemical and Biological Characterization of a Biosimilar Trastuzumab

**DOI:** 10.1155/2015/427235

**Published:** 2015-05-17

**Authors:** Carlos A. López-Morales, Mariana P. Miranda-Hernández, L. Carmina Juárez-Bayardo, Nancy D. Ramírez-Ibáñez, Alexis J. Romero-Díaz, Nelly Piña-Lara, Víctor R. Campos-García, Néstor O. Pérez, Luis F. Flores-Ortiz, Emilio Medina-Rivero

**Affiliations:** ^1^Unidad de Investigación y Desarrollo, Probiomed S.A. de C.V., Cruce de Carreteras Acatzingo-Zumpahuacán s/n, 52400 Tenancingo, MEX, Mexico; ^2^MUHC Research Institute, Royal Victoria Hospital, McGill University, 687 Pine Avenue West, Montreal, QC, Canada H3A 1A1

## Abstract

According to the World Health Organization, the incidence of malignant neoplasms and endocrine, blood, and immune disorders will increase in the upcoming decades along with the demand of affordable treatments. In response to this need, the development of biosimilar drugs is increasing worldwide. The approval of biosimilars relies on the compliance with international guidelines, starting with the demonstration of similarity in their physicochemical and functional properties against the reference product. Subsequent clinical studies are performed to demonstrate similar pharmacological behavior and to diminish the uncertainty related to their safety and efficacy. Herein we present a comparability exercise between a biosimilar trastuzumab and its reference product, by using a hierarchical strategy with an orthogonal approach, to assess the physicochemical and biological attributes with potential impact on its pharmacokinetics, pharmacodynamics, and immunogenicity. Our results showed that the high degree of similarity in the physicochemical attributes of the biosimilar trastuzumab with respect to the reference product resulted in comparable biological activity, demonstrating that a controlled process is able to provide consistently the expected product. These results also constitute the basis for the design of subsequent delimited pharmacological studies, as they diminish the uncertainty of exhibiting different profiles.

## 1. Introduction

Biopharmaceutical products containing chimeric, humanized, or fully human monoclonal antibodies (mAbs) are among the most successful and demanded therapies due to their highly specific mechanisms of action that result in an improvement of the patients' conditions and an increase in the survival rate, while minimizing the adverse side-effects when compared to other treatments [1]. Consequently, new manufacturing sites, process scale-ups as well as process improvements contribute to the well-known heterogeneity, naturally present in biotherapeutic products. For this purpose, the ICH Q5 E guideline provides the principles for assessing comparability of licensed biotechnological products subject to process changes throughout their life cycle [2].

In this sense, the approval of biosimilar products, which have been recognized not only as an alternative but as a necessity to increase health coverage and improve the quality of life of patients, follows a similar comparability scheme. International guidelines on biosimilarity [3–5] outline that the approval of biosimilars must rely on the demonstration of comparability towards the reference product, starting with an exhaustive physicochemical and biological characterization whose results will provide evidence to support the extent of additional clinical evaluation [6–8].

For this purpose, the proper identification of critical quality attributes (CQAs) that may impact on the pharmacokinetics, pharmacodynamics, and immunogenicity can be achieved through a deep knowledge of the chemical composition and the higher order structure of the active pharmaceutical ingredient (API) contained in the reference product, as well as the known relationships between specific attributes and biological functionality, anticipated by the biotechnological industry and the scientific community [9–17]. Furthermore, the ICH Q9 guideline highlights the need of evaluating the quality of a biopharmaceutical product based on a risk analysis that considers relevant attributes to the drug's safety and efficacy [18].

In this work we present a comparability study between a biosimilar trastuzumab and its reference product. Trastuzumab is a humanized monoclonal antibody targeted against the extracellular portion of the human epidermal growth factor receptor (HER2, p185), which is overexpressed in approximately 15 to 30% of the invasive breast cancer cases [19–22]. The chemical, physical, and functional properties closely related to its pharmacological behavior were identified through a risk analysis; then those CQAs were evaluated using several analytical techniques in an orthogonal approach that increases the reliability of the results obtained.

## 2. Materials and Methods

### 2.1. Materials

Biosimilar trastuzumab (440 mg powder for concentrate for solution for infusion) from Probiomed S.A. de C.V. (Mexico City, Mexico) and Herceptin (440 mg powder for concentrate for solution for infusion) from F. Hoffmann, La Roche Ltd. (Basel, Switzerland), were used for the comparability study.

### 2.2. Methods

#### 2.2.1. Physicochemical Properties

Primary sequences, verified from the whole-molecule exact masses and tryptic peptide mappings, were analyzed by reverse phase ultra-performance-liquid-chromatography coupled to a tandem quadrupole/time-of-flight mass spectrometer (RP-UPLC-MS/MS). Higher order structure was evaluated by differential scanning calorimetry (DSC), circular dichroism (CD), and fluorescence lifetime using the time correlated single photon counting technique (TCSPC). Charge heterogeneity of the whole, carboxypeptidase-digested, and papain-digested molecule was assessed either by capillary isoelectrofocusing (cIEF) or by cation exchange ultra-performance-liquid-chromatography (CEX-UPLC). Purity was determined by capillary gel electrophoresis under reducing (CGE-R) and nonreducing (CGE-NR) conditions and size exclusion ultra-performance-liquid-chromatography (SE-UPLC). Sample treatment and analysis conditions were performed as previously described for RP-UPLC-MS/MS, DSC, CD, CEX, CGE-R, and CGE-NR by Flores-Ortiz et al., 2014 [23]; TCSPC by Pérez Medina Martínez et al., 2014 [24]; cIEF by Espinosa-de la Garza et al. [25].

N-linked glycans were released from trastuzumab by enzymatic hydrolysis using PNGase F from New England Biolabs Inc. (Ipswich, MA) and then were labeled with 8-aminopyrene-1,3,6-trisulfonic acid (APTS) and analyzed by capillary zone electrophoresis (CZE) [26]. The electrophoretic separation was carried out in a PA 800 plus Analysis System from Beckman Coulter Inc. (Brea, CA) using an amine coated capillary of 50 *μ*m I.D. × 50.2 cm total length, with 40 cm effective length at 20°C. Laser induced fluorescence (LIF) detection was used at an excitation wavelength of 488 nm and emission band-pass filter of 520 nm. An orthogonal analysis was performed by hydrophilic interaction ultra-performance-liquid-chromatography (HILI-UPLC) after labeling with 2-aminobenzoic acid (2-AB) following a previously reported methodology [27].

### 2.3. Functional Properties

#### 2.3.1. Fc*γ*RIIIa Affinity by Isothermal Titration Calorimetry (ITC)

Affinity constants under equilibrium (*K*_*a*_) were obtained from a Nano ITC instrument (TA Instruments Inc.; New Castle, DE). 300 *μ*L of Fc*γ*RIIIa solutions at 5.0 *μ*M in PBS at pH 7.2 was titrated with continuous injections of 1.9 *μ*L trastuzumab solutions at 50 *μ*M in PBS at pH 7.2 until saturation at 25°C. NanoAnalyze Software v2.4.1 (TA Instruments Inc.; New Castle, DE) was used for the integration of heat signals and nonlinear regression analysis of the data.

#### 2.3.2. FcRn Affinity by BLI

Binding kinetics of trastuzumab to FcRn were determined using a Bio-Layer Interferometry (BLI) instrument, Octet QK384, from Pall ForteBio Corp. (Menlo Park, California). Biotinylated FcRn was immobilized to biosensors coated with streptavidin. Binding profiles were displayed by sensograms. Global kinetic analyses were determined using a 2 : 1 heterogeneous ligand model fit using R-linked analysis.

#### 2.3.3. HER2 Affinity Assay

HER2 expressing cells SK-BR-3 (ATCC HTB-30) were incubated in the presence of different concentrations of trastuzumab in McCoy-5A medium with 10% FBS for 2 h at 37°C. HRP-conjugated goat anti-human IgG was added to detect the trastuzumab–SK-BR-3 complex after 1 h of incubation at 37°C, using TMB as substrate for 30 min at room temperature. Absorption was measured at 450 nm. Test results were expressed as the relative percentage of the EC_50_ from the dose-response curve of the biosimilar trastuzumab with respect to the reference product.

#### 2.3.4. Antiproliferation Assay

BT-474 cells (ATCC HTB-20) were seeded in DMEM media with 10% FBS, 1% nonessential amino acids, and incubated at 37°C. Different concentrations of trastuzumab were added with further incubation for 8 days. Crystal violet was added to stain the cells for 15 min at room temperature followed by fixation with formaldehyde and water rising. Acetic acid aqueous solution (33% v/v) was added to remove the dye excess; absorbance was measured at 540 nm. Test results were expressed as the relative percentage of the EC_50_ from the dose-response curve of the biosimilar trastuzumab with respect to the reference product.

## 3. Results and Discussion

**Figure 1:**
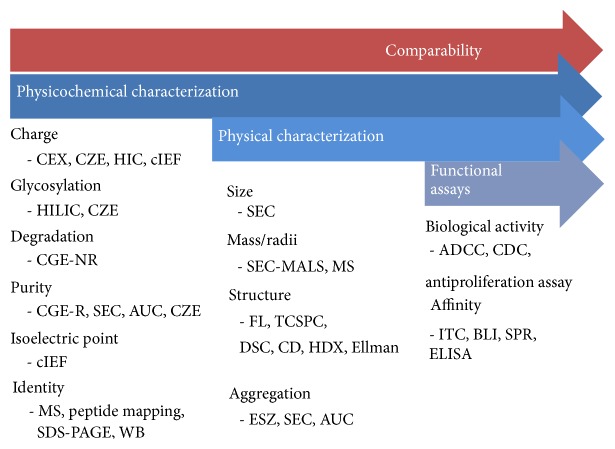
Characterization strategy performed for Trastuzumab-Probiomed.

Our characterization strategy (Figure 1) comprised a set of state-of-the-art analytical techniques planned for a hierarchical study of a biosimilar trastuzumab using an orthogonal approach. CQAs were identified using a risk analysis, considering each of the physicochemical and functional properties that may have an impact on efficacy (pharmacokinetics and pharmacodynamics) and safety (immunogenicity) of trastuzumab (Table 1) [9–17]. In this work, only certain methodologies were selected to depict a global overview of the characterization study. Hereafter, CQAs were classified by their physicochemical, physical, or biological nature and analyzed comparatively for a biosimilar trastuzumab (Trastuzumab-Probiomed) and its reference product.

### 3.1. Physicochemical Properties

**Figure 2:**
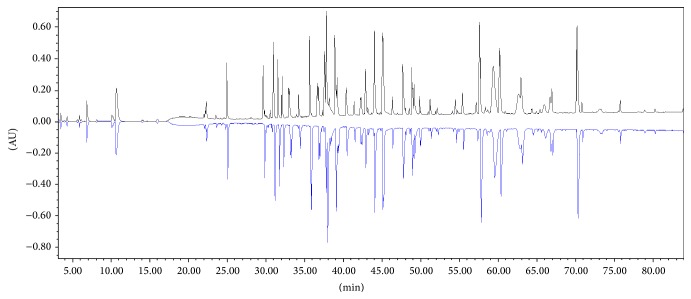
Mirror plot of peptide mapping chromatograms obtained from RP-UPLC-UV for Trastuzumab-Probiomed (upper) and the reference product (lower).

**Figure 3:**
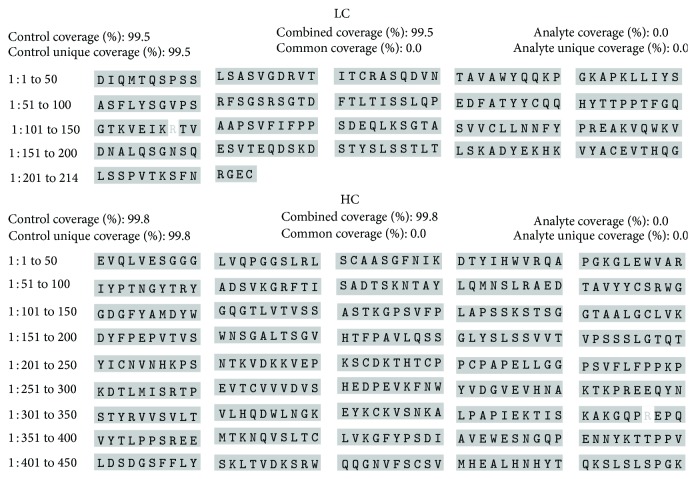
Sequence coverage of the heavy and light chains of Trastuzumab-Probiomed obtained from the MS/MS analysis.

**Figure 4:**
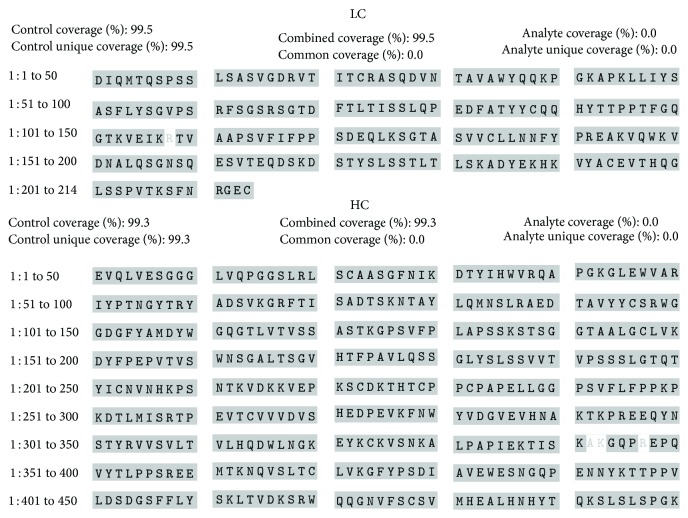
Sequence coverage of the heavy and light chains of the reference product obtained from the MS/MS analysis.

The identity of Trastuzumab-Probiomed towards the reference product was determined by the correspondence of their tryptic peptide mappings (Figure 2). MS/MS analysis verified the amino acid sequence of both products against the theoretical stated on the invention patent of trastuzumab [28], unveiling a sequence matching of 99.8% and 99.3% for the heavy chain and 99.5% and 99.5% for the light chain, for both Trastuzumab-Probiomed and the reference product, respectively (Figures 3 and 4). This correspondence was further confirmed by the analyses of the exact masses against the theoretical mass [28, 29] for both whole and deglycosylated molecules (Tables 2 and 3). The sequences coverage confirms that the amino acid sequence of Trastuzumab-Probiomed is identical to the reference product, while the <25 Da observed differences in intact masses for the whole molecule, below the expected width of the isotopic pattern distribution of a mAb, show in advance a comparable degree of heterogeneity, due to posttranslational modifications, in both products, ultimately producing an equivalent immunogenic response.

Regarding glycan microheterogeneity, which is known to contribute to the correct folding and stability of a mAb, it was analyzed by CZE and HILI-UPLC. Particularly, highly mannosylated and sialylated glycoforms are reported to alter a mAb half-life in blood and are linked to potential immunogenic responses; moreover effector functions can be altered due to the presence of highly mannosylated, bisected, and fucosylated glycoforms, as a consequence of charge or steric hindrances [10–12].

**Figure 5:**
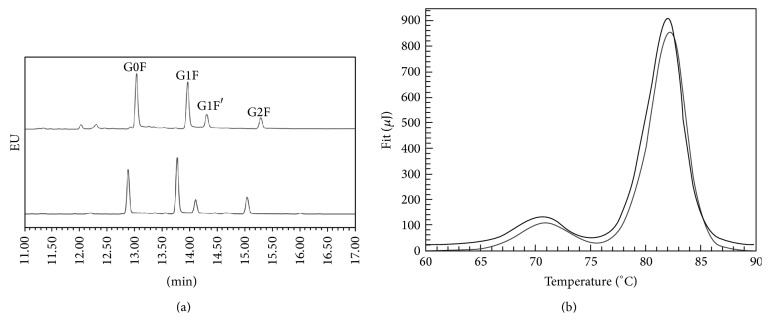
(a) Glycan profile for the reference product (upper) and Trastuzumab-Probiomed (lower). (b) Thermostability by DSC for the reference product (lower) and Trastuzumab-Probiomed (upper).

CZE analyses revealed that the glycan patterns of Trastuzumab-Probiomed and the reference product are comprised of the same principal glycoforms (Figure 5(a)), showing a mean relative abundance of galactosylated variants of 66.01% and 49.57% ± 6.18 (CI 95%) for Trastuzumab-Probiomed and the reference product, respectively, which is not expected to have an impact on the functional properties, since galactosylation has not been reported to alter the mechanisms of action of mAbs, as confirmed by the affinities and biological potency analyses discussed below. Further analysis by HILI-UPLC of the glycoforms identified as critical for PK, PD, or immunogenicity (Table 1) revealed comparable relative abundances of highly mannosylated variants, being 2.00 ± 0.10 (CI 95%) and 3.96 ± 0.45 (CI 95%) for Trastuzumab-Probiomed and the reference product, respectively, whereas the mean abundance for hybrid and sialylated variants was 4.75 ± 0.19 (CI 95%) and 0.27 ± 0.08 (CI 95%) for the reference product and 2.95 ± 0.15 (CI 95%) and 1.06 ± 0.14 (CI 95%) for Trastuzumab-Probiomed, respectively. These results confirm similarity of the critical glycoforms between Trastuzumab-Probiomed and the reference product; thus similar PK and PD profiles and no differential immunogenicity response are expected.

On the other hand, charge heterogeneity evaluated through cIEF analysis revealed that isoelectric points (pI) for the main isoform were 8.69 ± 0.00 (CI 95%) for Trastuzumab-Probiomed and 8.70 ± 0.01 (CI 95%) for the reference product, in accordance with the expected pI variations during manufacturing, no larger than 0.2 units [15, 16]. The observed isoform-abundance-weighted pI values confirmed similarity of charge heterogeneity among products, being 8.60 ± 0.01 (CI 95%) for Trastuzumab-Probiomed and 8.61 ± 0.01 (CI 95%) for the reference product. It has been reported that only changes in one pI unit can significantly alter the therapeutic activity of a mAb; thus the observed variation is not expected to affect the clinical behavior of Trastuzumab-Probiomed with respect to the reference product.

An orthogonal analytical technique for the evaluation of charge heterogeneity was CEX-UPLC, which revealed that the averaged abundances of the main, acidic, and basic isoforms were within the same order of magnitude for both products, being the mean values of 57.0%, 33.2%, and 9.8% (*n* = 3) for Trastuzumab-Probiomed and 62.5%, 27.3%, and 10.3% (*n* = 3) for the reference product, respectively. Furthermore, the results obtained after digestion with carboxypeptidase B showed also a comparable content of basic, acidic, and main isoforms among the two products, with a main relative content of 16.4%, 30.6%, and 53.0% (*n* = 3) for the reference product and 8.8%, 37.8%, and 53.4% (*n* = 3) for Trastuzumab-Probiomed, respectively.

After papain digestion, the mean abundance of basic isoforms in the reference product (*n* = 3) was 3.8% for the Fc fragment and 4.9% for the Fab fragment, whereas for Trastuzumab-Probiomed (*n* = 3) it was 4.2% for the Fc fragment and 6.5% for the Fab fragment. Regarding acidic isoforms, the mean abundance was 3.3% for the Fc fragment and 16.7% for the Fab fragment of the reference product, while for Trastuzumab-Probiomed it was 3.7% for the Fc fragment and 16.1% for the Fab fragment. Finally, the abundance of the Fc and Fab fragments was 26.7% and 44.6%, respectively, for the reference product, and for Trastuzumab-Probiomed the abundance of the Fc and Fab fragments was 25.7% and 43.8%, respectively.

Overall the results from cIEF and CEX-UPLC show that both products exhibit comparable charge heterogeneities, either as a whole molecule or as the fragments responsible for the recognition and effector functions of trastuzumab; thus no differences in functional activity should be expected.

CGE-NR and SE-UPLC results demonstrated that both products have a similar degree of purity (Tables 4 and 5) based on the relative content of monomer with respect to the presence of aggregates and other degraded or truncated isoforms. It is known that protein aggregation can induce immunogenicity; although a small amount of aggregates is expected, this amount is likely to increase due to stress conditions that a mAb may undergo during its manufacture, purification, formulation, and shelf-life [9, 30]. Aggregation may reveal new epitopes that potentially could stimulate the production of anti-drug antibodies (ADAs) resulting in the loss of activity, immunogenic reactions, or adverse effects during administration. Likewise, the presence of fragments or truncated forms coming from hydrolysis reactions could negatively impact on the safety and therapeutic effect of a mAb [31, 32]. The content of aggregates and truncated forms of Trastuzumab-Probiomed were lower than the limits established by the USP [29] and were comparable to the reference product; thus the risk of developing a different immunogenic response (differential immunogenicity) is diminished.

### 3.2. Physical Properties

**Figure 6:**
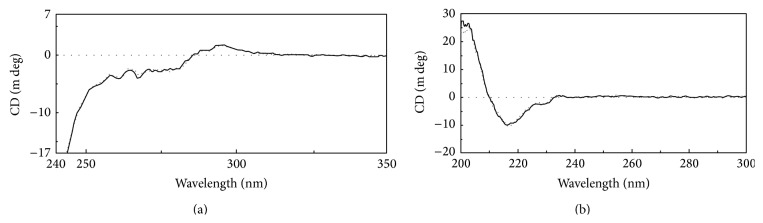
Analysis of the three-dimensional structure of trastuzumab by CD of Trastuzumab-Probiomed (solid line) and the reference product (dotted line) in both near-UV region (a) and far-UV region (b).

Since the functionality of trastuzumab is affected by its three-dimensional structure, which results from its primary sequence and posttranslational modifications that alter its size, mass, folding, and stability [8], we performed analyses to assess the spatial configuration of Trastuzumab-Probiomed compared to its reference product. Time correlated single photon counting analysis (TCSPC) was employed to evaluate the fluorescence lifetime (*τ*), which depends on the exposure of aromatic amino acids within the protein, thus demonstrating similarity when the results are obtained from comparative analyses [33–36]. TCSPC results showed that the averaged *τ* of Trastuzumab-Probiomed was 3.43*E* ^−09^  ± 1.39*E* ^−10^ s (CI 95%), while the averaged *τ* for the reference product was 3.49*E* ^−09^  ± 1.69*E* ^−11^ s (CI 95%). Regarding CD, the obtained spectrograms were superimposable in both near- and far-UV regions (Figure 6) suggesting that alpha helix, beta sheets, random coil, disulfide bonds, and aromatic amino acids are distributed in a comparable spatial arrangement. Finally, transition temperatures (*T*_*m*_) of Trastuzumab-Probiomed (*n* = 3) by DSC (Figure 5(b)) were 70.4°C, 79.1°C, 81.0°C, and 82.5°C, whereas for the reference product (*n* = 3) they were 70.5°C, 79.6°C, 81.2°C, and 82.7°C; for both products the CI at 95% was <0.02°C for all the temperatures. Collectively TCSPC, CD, and DSC determined that thermostability and secondary and tertiary structures of Trastuzumab-Probiomed were comparable to the reference product. In particular, thermostability results are indicative of a proper protein folding of both products in their respective formulation. This physicochemical and physical similarity is the major contributor to equivalent biological and functional responses.

### 3.3. Functional Properties

**Figure 7:**
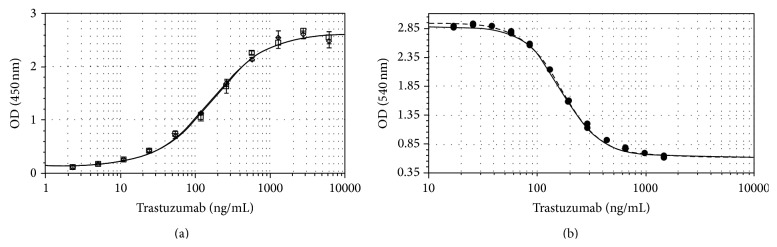
Comparison of* in vitro* activity between Trastuzumab-Probiomed and the reference product. (a) Curve of binding affinity to HER2; (b) potency curve obtained from the antiproliferation assay; the solid line corresponds to Trastuzumab-Probiomed, while the dashed line corresponds to the reference product.

The relative affinity of Trastuzumab-Probiomed towards its target molecule, HER2 (Figure 7(a) and Table 7), was evaluated with respect to the reference product, resulting in an averaged relative affinity of 97.7%. Thus, it is expected that Trastuzumab-Probiomed can exert its activity through the reported mechanisms of action, including HER2 downregulation, prevention of the heterodimer formation, initiation of Gl arrest, induction of p27, and prevention of HER2 cleavage [37].

The main mechanisms of action rely on the affinity of the Fc fragment of trastuzumab towards Fc*γ* receptors. For instance, Fc*γ*RIIIa present on effector cells such as macrophages, monocytes, and natural killer cells activates and induces ADCC mechanism against HER2-positive cells [37, 38]. Binding affinities towards Fc*γ*RIIIa were evaluated by ITC, being the averaged affinity constants (*K*_*a*_) of 2.61 ± 0.54*E* ^+06^ M^−1^ for Trastuzumab-Probiomed and 2.48 ± 0.30*E* ^+06^ M^−1^ for the reference product (Table 6). Likewise, the mean dissociation constants (*K*_*D*_) to FcRn, which regulates IgG catabolism, were determined by BLI as 2.58*E* ^−07^ M ± 1.02*E* ^−07^ M (CI 95%) for Trastuzumab-Probiomed with a relative binding affinity of 114.3% (*n* = 3) with respect to the reference product. Based on these results no differences in the half-life in blood are expected.

The overall* in vitro* activity was tested between Trastuzumab-Probiomed and the reference product with an antiproliferation assay (Figure 7(b)), which demonstrated that both products have the same potency to deplete HER2-positive cells, being the mean relative potencies towards the reference product of 105%, 103%, and 110% for three different batches of Trastuzumab-Probiomed, demonstrating that similarity on physicochemical and physical critical quality attributes results in a comparable biological potency.

## 4. Conclusions

During the development of a biosimilar, an extended characterization of its physicochemical and functional properties is required to gain a strong knowledge of its CQAs. This allows the establishment of in-process control strategies and quality specifications to ensure batch-to-batch consistency in order to obtain the desired product, despite the fact that it has been produced using a different manufacturing process with respect to the reference product. In addition, the use of orthogonal methods during a comparability study provides a global overview of the molecule and confirms the observed results on relevant modifications. Here, it was demonstrated that similarity between the critical physicochemical attributes resulted in comparable biological properties.

The observed physicochemical and functional similarity between products, as part of the totality-of-the-evidence scheme, will determine the extent of upcoming nonclinical and clinical studies, considering that it diminishes the uncertainty of exhibiting different pharmacological profiles.

## Figures and Tables

**Table 1:** Impact of CQAs on safety and efficacy.

Attribute	Pharmacodynamics	Pharmacokinetics	Immunogenicity

Sequence	Nonspecific	Nonspecific	Determined by the sequence variation against endogenous domains [9] Differential response due to sequence modifications for distinct batches or processes

Higher order structure	Nonspecific	Nonspecific	Determined by molecular weight and structure complexity [9]

Glycosylation profile	Fucosylated, highly mannosylated, and sialylated variants could alter *in vivo *efficacy [10–12]	Highly mannosylated variants show higher clearance Sialylated variants show lower clearance [10–12]	Sialic acid residues can hide antigenic determinants [9, 10] Highly mannosylated, hybrid, and nonglycosylated variants are prone to elicit immunogenicity

Charge heterogeneity	Effector functions altered if pI differences are >1 unit [10, 14, 15]	Major differences alter volume of distribution and clearance [10, 14, 15]	Acidic variants are prone to elicit immunogenicity [9]

Aggregates	Lower biological activity [11]	Less subcutaneous absorption and lower bioavailability [11]	ADAs presence [10]

Fc*γ*RI affinity	Affects endocytosis, antigen presentation	Not determined	Not determined
Fc*γ*RII affinity	ADCC, phagocytosis [17]		
Fc*γ*RIII affinity	Higher affinity to specific variants [11, 12] Affects endocytosis, antigen presentation, ADCC, phagocytosis [17]		

FcRn affinity	Not determined	Lower affinity to acidic variants Lower affinity for oxidized methionine Not expected measurable differences in variants with 3- to 4-fold changes in FcRn affinity [16]	Not determined

**Table 2:** Whole-molecule exact masses by MS.

Product	Batch	G0/G0F	G0F/G0F	G0F/G1F	G1F/G1F	G1F/G2F	G2F/G2F
Averaged theoretical	—	147911.76	148057.91	148220.05	148382.19	148544.33	148706.46
Reference product	B3417B010	147907.81	148061.92	148220.20	148378.84	148536.86	148692.48
	B3433B010	147897.68	148058.03	148218.21	148377.11	148534.93	148690.07
	N3477B021	147899.82	148058.00	148218.10	148377.32	148535.39	148690.95

Trastuzumab-Probiomed	TZPP12001	147901.19	148057.88	148218.18	148378.54	148537.97	148695.60
	TZPP12002	147900.45	148057.89	148217.99	148378.23	148537.59	148694.92
	TZPP12003	147898.55	148057.58	148217.49	148377.84	148537.14	148694.42

**Table 3:** Deglycosylated molecule exact masses by MS.

Product	Batch	Mass (Da)
Theoretical	—	145167.36

Reference product	B3417B010	145167.47
	B3433B010	145167.36
	N3477B021	145167.16

Trastuzumab-Probiomed	TZPP12002	145167.53
	TZPP12001	145167.08
	TZPP12003	145167.69

**Table 4:** Monomer content of trastuzumab by SE-UPLC and CGE-NR. Variation is presented as confidence intervals at 95% (*n* = 3).

Product	Batch	SE-UPLC (%)	CGE-NR (%)
Trastuzumab-Probiomed	TZPP11002	99.6 ± 0.0	92.3 ± 0.3
	TZPP12001	98.9 ± 0.0	90.8 ± 1.1
	TZPP11001	99.4 ± 0.0	96.6 ± 0.4

Reference product	N3597B013	98.9 ± 0.1	92.8 ± 0.6
	N35973	99.7 ± 0.0	93.5 ± 0.7
	B34310	99.5 ± 0.0	93.1 ± 0.4

**Table 5:** Relative abundance of trastuzumab subunits by CGE-R. Variation is presented as confidence interval at 95% (*n* = 3).

Product	Batch	HC %	NGHC %	LC %
Trastuzumab-Probiomed	TZPP12001	66.18 ± 0.16	0.57 ± 0.06	32.89 ± 0.19
	TZPP12002	64.46 ± 0.47	0.58 ± 0.02	34.59 ± 0.32
	TZPP12003	65.45 ± 0.97	0.50 ± 0.05	33.53 ± 1.08

Reference product	B3393B019	65.14 ± 0.25	0.57 ± 0.03	33.93 ± 0.13
	B3417B010	66.02 ± 0.27	0.52 ± 0.09	33.04 ± 0.25
	B3430	66.40 ± 0.25	0.63 ± 0.01	32.46 ± 0.16

HC: heavy chain, NGHC: nonglycosylated heavy chain, and LC: light chain.

**Table 6:** Affinity of trastuzumab to Fc*γ*RIIIa.

Product	Batch	Affinity constant (*K*_*a*_) to Fc*γ*RIIIa (M^−1^)
Trastuzumab-Probiomed	TZPP14001	2.71*E* + 06
	TZPP12002	2.86*E* + 06
	TZPP12003	2.25*E* + 06

Reference product	N35893	2.66*E* + 06
	N35812	2.48*E* + 06
	N36003	2.31*E* + 06

**Table 7:** Binding affinity of trastuzumab to the epidermal growth factor receptor (HER2).

Product	Batch	Relative affinity (%)
Trastuzumab-Probiomed	TZPP11001	98
	TZPP12004	98
	TZPP12003	97

Reference product	N3654	119
	N36263	111
	N36443	112
